# Impact of Hepatic Artery Diameter on Immediate Postoperative Graft Function and Hemodynamic Profiles in Living-Donor Liver Transplantation: A Retrospective Cohort Study

**DOI:** 10.3390/medicina62102006

**Published:** 2026-10-16

**Authors:** Il Young Ahn, In Ho Jung, Suk Won Suh, Shin Hyuk Kang, Soo Hyun Woo, Han Koo Kim

**Affiliations:** 1 Department of Plastic and Reconstructive Surgery, Chung-Ang University Hospital, Chung-Ang University College of Medicine, 102 Heukseok-ro, Dongjak-gu, Seoul 06973, Republic of Korea; ahnilyo@gmail.com (I.Y.A.); 07707@cauhs.or.kr (I.H.J.); kangshinhyeok@cau.ac.kr (S.H.K.); shwoops@cauhs.or.kr (S.H.W.); 2 Department of Surgery, Chung-Ang University College of Medicine, Seoul 06973, Republic of Korea; bumboy1@cau.ac.kr

**Keywords:** living-donor liver transplantation, hepatic artery reconstruction, microsurgery, multidisciplinary approach, linear mixed model

## Abstract

*Background and Objectives*: Reconstruction of small-caliber hepatic arteries (HAs) in living-donor liver transplantation (LDLT) is technically challenging. We evaluated clinical and hemodynamic outcomes according to intraoperative HA diameter in a retrospective single-center cohort of 49 patients who underwent LDLT between 2017 and 2026. *Materials and Methods*: Patients were stratified into Group 1 (≤2.0 mm, *n* = 28), Group 2 (2.5 mm, *n* = 6), and Group 3 (≥3.0 mm, *n* = 15). Perioperative variables, laboratory markers of graft function for 14 days, and Doppler hemodynamic parameters for 7 days were analyzed using linear mixed models; HA diameter was also analyzed as a continuous variable, with adjustment for graft-to-recipient weight ratio and MELD-Na score. *Results*: Baseline and surgical variables did not differ significantly, and neither group nor group × time effects were significant for any outcome. Per 1-mm increase in diameter, AST and ALT ratios were 1.01 (95% CI 0.83–1.23) and 1.06 (0.79–1.42). Early allograft dysfunction occurred in 5 patients (indeterminate in 3). No hepatic artery thrombosis, arterial re-intervention, retransplantation or 90-day death was documented; 14 patients (28.6%) required biliary intervention. *Conclusions*: In this small cohort, a smaller donor HA diameter was not associated with worse early biochemical or Doppler trajectories, but the estimates were imprecise and cannot establish equivalence or vascular safety.

## 1. Introduction

Anatomical reconstruction of the hepatic artery (HA) remains one of the most critical and technically demanding steps in living-donor liver transplantation (LDLT). Due to the inherently small diameter and complex anatomical variations of the donor and recipient arteries, HA anastomosis requires precise microvascular techniques to prevent severe vascular complications, such as hepatic artery thrombosis (HAT) [1]. Although advances in microsurgical instruments have significantly improved patency rates, reconstructing small-caliber HAs remains a major technical challenge for transplant surgeons, often increasing overall surgical time and technical difficulty [2]. To address these technical hurdles, a multidisciplinary approach involving plastic surgeons specializing in microsurgery for HA reconstruction has been actively implemented at our institution, optimizing the macro- and micro-surgical components of the procedure [3,4].

While numerous studies have investigated the long-term outcomes of liver transplantation, assessing long-term graft survival in relation to HA size introduces various confounding variables, including chronic rejection, biliary strictures, and systemic metabolic changes [5,6]. Consequently, focusing on the immediate postoperative period may provide a more direct reflection of the technical success of the microvascular anastomosis [7,8]. Short-term graft function, early hemodynamic parameters, and immediate perioperative outcomes may serve as early surrogates for evaluating the quality of initial arterial inflow and the efficacy of the microsurgical technique [9].

To date, few clinical studies have specifically evaluated the short-term outcomes of LDLT based on the intraoperative diameter of the hepatic artery using daily longitudinal data. Therefore, this study aims to investigate whether the size of the hepatic artery influences perioperative variables (such as total operative time and length of hospital stay) [10], early graft function monitored via daily laboratory markers (AST, ALT, total and direct bilirubin, and INR) [8], and immediate hemodynamic profiles evaluated through daily Doppler ultrasonography (PSV, RI, and SAT delay) [9]. Through this objective comparison, we sought to determine whether a smaller HA size adversely affects early graft function and hemodynamics.

## 2. Materials and Methods

### 2.1. Patient Population and Selection Criteria

This retrospective cohort study initially reviewed data from patients who underwent LDLT at Chung-Ang University Hospital between March 2017 and March 2026. A total of 53 patients were originally screened. Among them, three patients were excluded because their intraoperative HA diameters were not documented in the medical records. One patient was excluded due to an intraoperative cardiac arrest of unknown etiology, which prevented the completion of standard postoperative follow-ups.

A final cohort of 49 patients was included in the analysis. Based on the intraoperative measurements recorded in the EMR, the hepatic artery diameters were documented in discrete intervals of 0.5 mm. Patients were stratified into three groups:

• Group 1 (≤2.0 mm): *n* = 28
• Group 2 (2.5 mm): *n* = 6
• Group 3 (≥3.0 mm): *n* = 15

All 28 patients in Group 1 had a recorded diameter of exactly 2.0 mm. Group 3 comprised 13 patients with 3.0 mm, one with 4.0 mm and one with 5.0 mm. The study protocol approved by the IRB specified donor HA diameter as the exposure but did not define cut-off values; the three categories were defined after data collection according to the recorded 0.5-mm values. A diameter of 2.0 mm or less has traditionally been regarded as a small-caliber HA in LDLT [2], and the 2.5-mm values were kept as a separate intermediate category. All grafts were right-lobe grafts.

### 2.2. Surgical Technique and Perioperative Variables

Macro-surgical procedures, including graft procurement and recipient hepatectomy, were performed by the transplantation surgery team. Microscopic HA anastomosis was performed by the plastic surgery team using standardized microvascular techniques under a surgical microscope. All arterial anastomoses were performed using interrupted sutures with 9-0 or 10-0 monofilament nylon (Ethilon), depending on the caliber and tissue quality of the hepatic artery.

The external diameter of the donor HA was measured at its intended anastomotic end by the plastic surgery microsurgeon, under the operating microscope, immediately before the anastomosis was started. A stainless steel double-ended vessel measuring gauge (GEM2749; Synovis Micro Companies Alliance, Inc., Birmingham, AL, USA) was placed against the vessel and the diameter was read by visual comparison. Diameters were recorded in 0.5-mm increments, because finer differences could not be distinguished consistently with this method. Papaverine was applied topically and repeatedly during the microvascular procedure; its timing relative to the measurement was not recorded.

Perioperative clinical outcomes were extracted from the electronic medical records (EMR). These included total operative time, plastic surgery participation time (the time of plastic surgery team participation as recorded in the operative records, including bench work; when more than one session was recorded, durations were summed), total length of hospital days (HD), post-operative days (POD) until discharge, ICU stay duration (days from transplantation to transfer to the general ward), harvested liver graft weight, and recipient preoperative body weight. The GRWR was calculated as graft weight (g)/[recipient body weight (kg) × 10] and expressed as a percentage. The preoperative MELD-Na score was calculated with the 2016 United Network for Organ Sharing formula from the serum creatinine, sodium, total bilirubin and INR values measured closest to surgery; creatinine was set to 4.0 mg/dL for patients receiving hemodialysis.

### 2.3. Postoperative Monitoring

• Serum Laboratory Tests: Serum levels of AST, ALT, total bilirubin, direct bilirubin, and INR were measured daily for 14 days postoperatively.
• Doppler Ultrasonography: Color Doppler ultrasonography was performed daily for the first 7 postoperative days to evaluate the patency and flow dynamics of the reconstructed HA. Quantitative parameters collected included the RI, PSV, and SAT delay.
• Clinical outcomes: Early allograft dysfunction (EAD) was defined at the patient level according to Olthoff et al. [11] as AST or ALT > 2000 IU/L within the first 7 postoperative days, total bilirubin ≥ 10 mg/dL on POD 7, or INR ≥ 1.6 on POD 7. Patients with a missing POD 7 value who met no other criterion were classified as indeterminate, not as EAD-negative. The medical records of all 49 patients were reviewed for the first 90 days after transplantation for death, retransplantation and any unplanned reoperation or therapeutic intervention. Hepatic artery thrombosis (HAT) was defined as arterial occlusion confirmed by CT angiography, angiography or reoperation. Interventions were classified as HA-related (HA re-anastomosis, thrombectomy, or endovascular treatment for HAT or stenosis), biliary (percutaneous drainage of a biloma, percutaneous transhepatic biliary drainage, endoscopic retrograde cholangiopancreatography or surgical repair for bile leakage or stricture), or other. Diagnostic procedures and simple paracentesis or thoracentesis were not counted, and the cause of biliary complications (anastomotic or ischemic) was not classified.

### 2.4. Statistical Analysis

Statistical analyses were performed using jamovi (version 2.6; The jamovi project) with the GAMLj3 module. Baseline demographics and perioperative continuous variables—such as total operative time, plastic surgery participation time, recipient weight, GRWR, MELD-Na score, HD, POD, ICU stay, and liver graft weight—were compared among the three groups using Welch’s one-way analysis of variance (ANOVA), except for the MELD-Na score, which was skewed and was compared using the Kruskal–Wallis test. Categorical variables were analyzed using the Chi-square test or Fisher’s exact test.

To analyze the daily longitudinal data of graft functions (AST, ALT, total bilirubin, direct bilirubin, and INR from the preoperative baseline to POD 14) and hemodynamic markers (PSV and RI from POD 1 to POD 7), an LMM was utilized. The LMM was selected to account for the unbalanced sample sizes across the three groups, handle occasional missing data points on specific postoperative days, and manage the intra-subject correlation among repeated measurements. In the model, the main effects of the HA size group and time (days, as a categorical factor), as well as their interaction effect (group × time), were specified as fixed effects, while individual patients were treated as random effects (random intercept; compound-symmetry structure). Random slopes for time were not included because time had 7–16 categorical levels and such a structure could not be estimated reliably in 49 patients. Models were fitted by restricted maximum likelihood (REML), fixed effects were tested with F tests using Satterthwaite degrees of freedom, and simple contrasts (each level versus the first, centered) were used for group and time. The omnibus group and group × time tests were the primary tests. To evaluate differences among the groups at each specific time point, simple main effects analysis was performed within the framework of the LMM. Group differences at individual time points were directly assessed using the parameter estimates derived from the model. These time-point-specific comparisons were exploratory; they are reported with unadjusted *p* values and with Holm adjustment within each outcome across all time points and group pairs (48 comparisons for each laboratory outcome and 21 for each Doppler outcome). Cross-sectional comparisons were performed daily to evaluate the proportion of patients presenting with SAT delay (SAT of 80 ms or more) among the cohorts, using Fisher’s exact test; patients without an SAT measurement on a given day were excluded from that day’s denominator. These daily tests were not adjusted for multiplicity and are considered exploratory. A *p*-value < 0.05 was considered statistically significant.

The following sensitivity analyses were added during peer review and were not prespecified. First, measured HA diameter (mm) was entered as a continuous covariate in place of group, with an HA diameter × time interaction; the diameter coefficient represents the effect per 1-mm increase averaged over time points. A model with a common diameter slope (no interaction) was also fitted. Second, GRWR and then the preoperative MELD-Na score were added as covariates to the group-based and continuous models; graft weight and recipient weight were not added separately because they define GRWR. No further covariates were added because of the limited number of patients. Third, because residual Q–Q and residual-versus-fitted plots showed right-skewed residuals, all models were refitted after natural-log transformation of the outcome (all values were positive); log-scale effects are reported as ratios (exp[B]) with 95% confidence intervals (CIs). Holm adjustment across the seven outcomes was applied to the continuous diameter effects. The influence of the two patients with the largest diameters (4.0 and 5.0 mm) was examined by refitting the continuous models without them.

The number of observations decreased after the first postoperative week. Reasons for individual missing measurements were not systematically recorded. LMM estimates are unbiased if data are missing at random given the observed data; this assumption could not be verified.

Holm-adjusted *p* values were calculated from the unadjusted *p* values. During the revision, Claude (Claude Opus 5.5, Anthropic) was used as an assistive tool for checking data entry and preparing the statistical analyses; all reported analyses were conducted and verified by the authors.

### 2.5. Ethics Approval and Informed Consent

This study was approved by the Chung-Ang University Hospital Institutional Review Board (approval code: 2604-032-19625; approval date: 7 May 2026) and was conducted in accordance with Declaration of Helsinki provisions.

The requirement for informed consent was waived by the Institutional Review Board owing to the retrospective nature of the study.

## 3. Results

### 3.1. Baseline Demographics and Underlying Etiologies

The baseline demographic characteristics and underlying etiologies of the 49 included patients are summarized in Table 1. No statistically significant differences in baseline characteristics were detected among the three groups.

The cohort included 36 male patients (73.5%), with 21 patients in Group 1 (≤2.0 mm), 4 in Group 2 (2.5 mm), and 11 in Group 3 (≥3.0 mm). The mean age of the entire patient population was 57.1 ± 10.6 years, and no statistically significant age differences were observed among the cohorts (55.9 ± 10.8 years in Group 1 vs. 58.0 ± 8.9 years in Group 2 vs. 58.8 ± 11.0 years in Group 3).

Alcoholic liver disease (either alone or combined with hepatocellular carcinoma) was the primary indication for LDLT, accounting for 57.1% (*n* = 28) of the total cohort. Specifically, isolated alcoholic cirrhosis was documented in 14, 1, and 6 patients across Groups 1, 2, and 3, respectively. Isolated hepatocellular carcinoma (HCC) was present in 16 patients (32.7%), followed by a combination of both alcoholic liver disease and HCC in 7 patients (14.3%). Viral hepatitis was identified in a subset of patients, including 12 cases of hepatitis B virus (HBV) infection (*n* = 6, 2, and 4 in Groups 1, 2, and 3, respectively) and 2 cases of hepatitis C virus (HCV) infection (both in Group 1).

### 3.2. Perioperative Outcomes and Clinical Variables

The perioperative outcomes and clinical variables among the three groups stratified by the intraoperative HA diameter are summarized in Table 2. There were no statistically significant differences among the groups across all evaluated parameters.

The total operation time was 626.75 ± 86.51 min in Group 1 (≤2.0 mm), 552.67 ± 81.33 min in Group 2 (2.5 mm), and 629.07 ± 123.02 min in Group 3 (≥3.0 mm) (*p* = 0.170). The plastic surgery participation time was 93.93 ± 52.01 min, 70.67 ± 31.21 min, and 87.20 ± 53.37 min, respectively (*p* = 0.384).

The duration of intensive care unit (ICU) stay was 8.89 ± 6.59 days for Group 1, 6.67 ± 1.51 days for Group 2, and 6.13 ± 1.06 days for Group 3 (*p* = 0.123). There were no significant differences in total hospital days (52.18 ± 61.38 vs. 35.00 ± 28.04 vs. 34.87 ± 18.92 days, *p* = 0.419) or postoperative days (POD) until discharge (33.50 ± 38.08 vs. 29.17 ± 20.22 vs. 25.47 ± 8.34 days, *p* = 0.562).

The harvested liver graft weight was 683.84 ± 137.62 g in Group 1, 755.83 ± 203.80 g in Group 2, and 770.80 ± 137.33 g in Group 3 (*p* = 0.181).

All grafts were right-lobe grafts. Recipient preoperative weight (63.29 ± 12.71, 68.48 ± 11.80 and 63.65 ± 16.07 kg; *p* = 0.644) and GRWR (1.12 ± 0.32%, 1.11 ± 0.26% and 1.28 ± 0.39%; *p* = 0.403) did not differ significantly among Groups 1, 2 and 3. A GRWR below 0.8% was present in 5, 1 and 1 patients, respectively. The preoperative MELD-Na score also did not differ significantly (median 14.0, 10.5 and 11.0; *p* = 0.727). One patient in Group 3 was receiving hemodialysis before transplantation, and two patients in Group 3 underwent plasmapheresis before transplantation, which may have lowered their preoperative bilirubin and INR values and thus their MELD-Na scores (Table 2).

### 3.3. Postoperative Laboratory Findings

Daily longitudinal serum levels of aspartate aminotransferase (AST), alanine aminotransferase (ALT), total and direct bilirubin, and the international normalized ratio (INR) were evaluated from the pre-operative baseline to post-operative day (POD) 14.

#### 3.3.1. Serum Aspartate Aminotransferase (AST) Alterations

**Figure 1:**
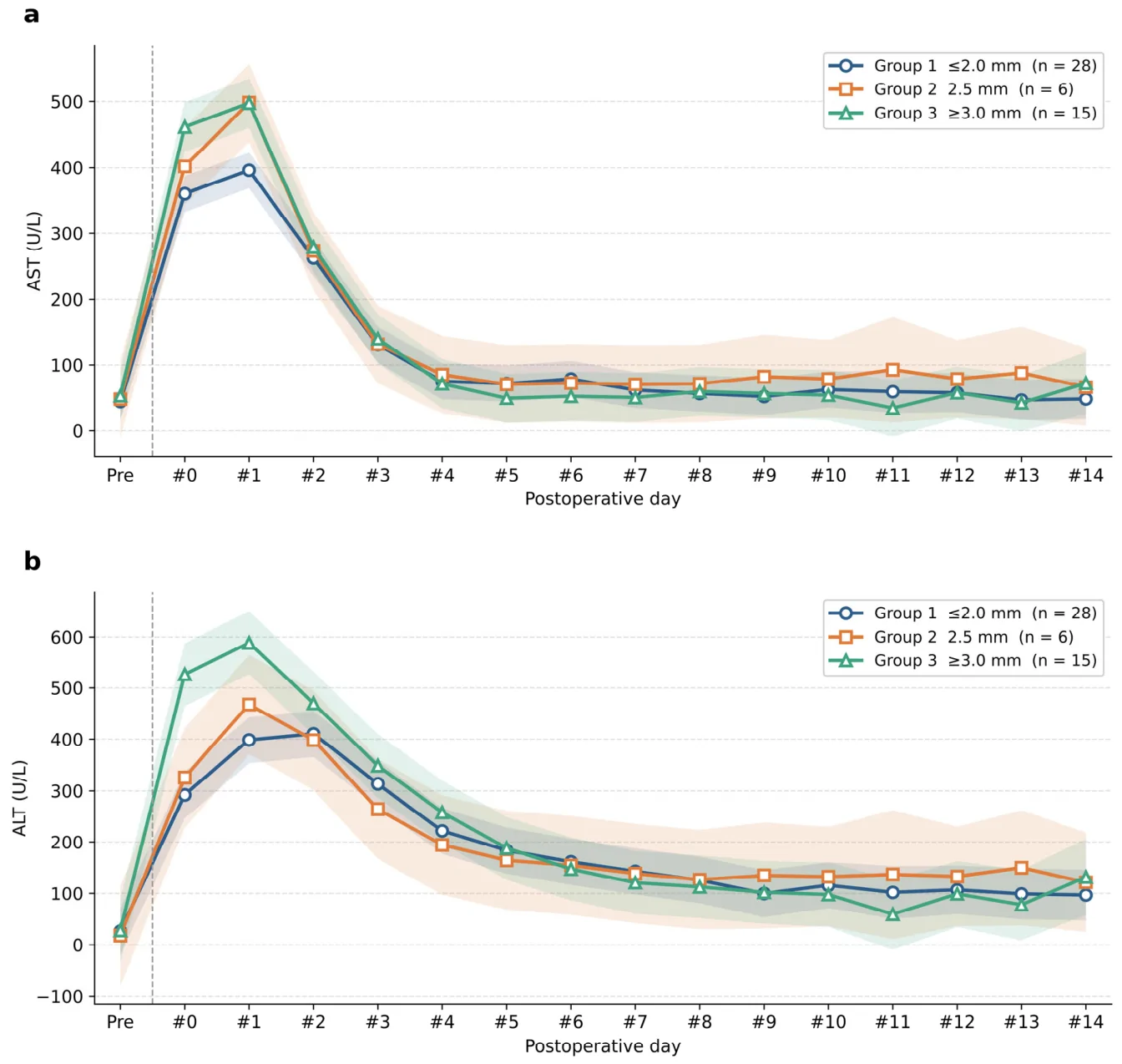
Postoperative serum aminotransferase levels by hepatic artery (HA) diameter group. (**a**) aspartate aminotransferase (AST) and (**b**) alanine aminotransferase (ALT) from the preoperative period through postoperative day (POD) 14. Lines represent estimated marginal means from the unadjusted, raw-scale group-based linear mixed models, and shaded areas represent standard errors; *n* is the number of patients in each group. No between-group difference at an individual time point remained significant after Holm adjustment (Supplementary Tables S1 and S2).

The chronological changes in serum AST levels for the three groups are presented in Supplementary Table S1 and illustrated in Figure 1a. Baseline pre-operative AST levels showed no significant differences among the groups (*p* = 0.981). Following liver transplantation, AST levels peaked on POD #1 across all cohorts.

Although the overall simple main effects analysis across the three groups did not reach statistical significance at any single time point (Supplementary Table S1; e.g., POD #0, *p* = 0.091; POD #1, *p* = 0.052), exploratory pairwise comparisons derived from the model’s parameter estimates showed nominal differences between specific cohorts. On POD #0 and POD #1, AST levels in Group 1 (≤2.0 mm) were lower than those in Group 3 (≥3.0 mm) (POD #0: 359.9 ± 27.4 U/L vs. 461.5 ± 37.4 U/L, unadjusted *p* = 0.029; POD #1: 395.9 ± 27.4 U/L vs. 497.5 ± 37.4 U/L, unadjusted *p* = 0.029); neither difference remained significant after Holm adjustment. No significant differences were observed between Group 1 and Group 2, or between Group 2 and Group 3 at these time points.

Subsequently, mean AST levels decreased in all three groups. By POD #4, the mean levels were below 100 U/L in all cohorts (75.3 ± 27.4 U/L, 85.3 ± 59.1 U/L, and 71.6 ± 37.4 U/L, respectively), with no statistically significant differences between the groups from POD #2 to POD #14.

The Linear Mixed Model (LMM) analysis across the 14-day period demonstrated a significant time effect (Table 3, *p* < 0.001). There was no significant main effect for the hepatic artery size group (group effect, *p* = 0.758) and no significant group-by-time interaction (group × time interaction, *p* = 0.995).

#### 3.3.2. Serum Alanine Aminotransferase (ALT) Alterations

The chronological changes in serum ALT levels for the three groups are presented in Supplementary Table S2 and Figure 1b. Baseline pre-operative ALT levels showed no significant differences among the groups (*p* = 0.996). Following transplantation, ALT levels peaked between POD #1 and POD #2 across all cohorts.

In exploratory simple main effects analysis, unadjusted differences among the groups were observed on POD #0 (overall *p* = 0.009) and POD #1 (overall *p* = 0.046) (Supplementary Table S2). Localized comparisons showed that ALT levels in Group 1 (≤2.0 mm) were lower than those in Group 3 (≥3.0 mm) on POD #0 (292.4 ± 44.9 U/L vs. 526.5 ± 61.3 U/L, unadjusted *p* = 0.002) and POD #1 (398.4 ± 44.9 U/L vs. 588.4 ± 61.3 U/L, unadjusted *p* = 0.013); neither difference remained significant after Holm adjustment (smallest adjusted *p* = 0.11). No significant differences were observed between Group 1 and Group 2, or between Group 2 and Group 3 at these time points. From POD #2 onward, ALT levels decreased across all cohorts, with no statistically significant differences observed among the groups at any subsequent time point up to POD #14.

The LMM analysis across the 14-day observation period demonstrated a significant time effect (Table 3, *p* < 0.001). There was no significant main effect for the hepatic artery size group (group effect, *p* = 0.837) and no significant group-by-time interaction (group × time interaction, *p* = 0.708).

#### 3.3.3. Serum Total and Direct Bilirubin Alterations

**Figure 2:**
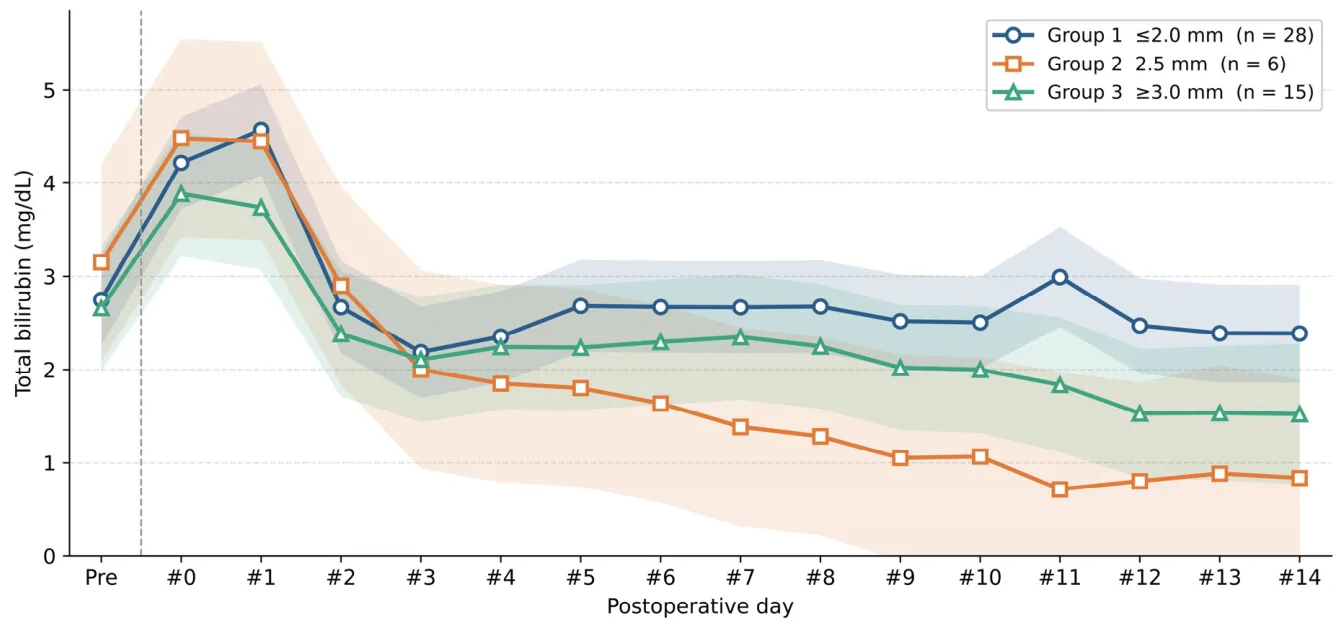
Postoperative serum total bilirubin levels by hepatic artery (HA) diameter group from the preoperative period through postoperative day (POD) 14. Lines represent estimated marginal means from the unadjusted, raw-scale group-based linear mixed models, and shaded areas represent standard errors; *n* is the number of patients in each group.

The longitudinal changes in serum total and direct bilirubin levels are detailed in Supplementary Table S3 and Figure 2, and Supplementary Table S4 and Supplementary Figure S1, respectively. Baseline pre-operative total and direct bilirubin levels showed no significant differences among the groups (total bilirubin: *p* = 0.925; direct bilirubin: *p* = 0.801).

Following transplantation, both total and direct bilirubin levels peaked between POD #0 and POD #1 across all groups. On POD #1, total bilirubin was 4.571 ± 0.492 mg/dL in Group 1 (≤2.0 mm), 4.450 ± 1.062 mg/dL in Group 2 (2.5 mm), and 3.740 ± 0.672 mg/dL in Group 3 (≥3.0 mm), with no significant difference among the groups (*p* = 0.602). Simple main effects analyses demonstrated no statistically significant differences among the three groups at any individual time point from POD #2 to POD #14 for either total or direct bilirubin (all daily *p* > 0.05).

The LMM analysis for total bilirubin demonstrated a significant time effect (Table 3, *p* < 0.001), but no significant main effect for the hepatic artery size group (group effect, *p* = 0.547) and no significant group-by-time interaction (group × time interaction, *p* = 0.923). For direct bilirubin, the model revealed no significant group effect (*p* = 0.574), no significant time effect (*p* = 0.172), and no significant group-by-time interaction (*p* = 0.948).

#### 3.3.4. Serum International Normalized Ratio (INR) Alterations

**Figure 3:**
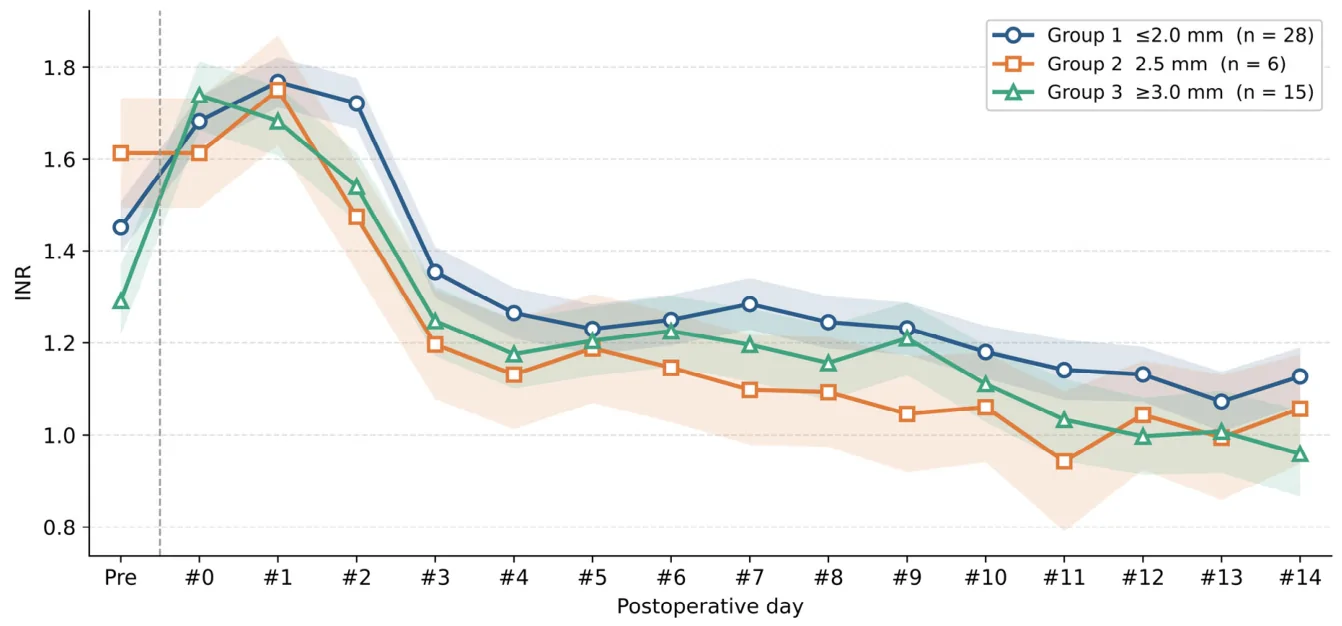
Postoperative international normalized ratio (INR) by hepatic artery (HA) diameter group from the preoperative period through postoperative day (POD) 14. Lines represent estimated marginal means from the unadjusted, raw-scale group-based linear mixed models, and shaded areas represent standard errors; *n* is the number of patients in each group.

The longitudinal changes in serum INR levels are presented in Supplementary Table S5 and Figure 3. The overall pre-operative baseline INR difference among the groups was not statistically significant (*p* = 0.055). In an exploratory pairwise comparison, preoperative INR was nominally higher in Group 2 (2.5 mm) than in Group 3 (≥3.0 mm) (1.613 ± 0.119 vs. 1.292 ± 0.075; unadjusted *p* = 0.024; not significant after Holm adjustment).

Following transplantation, INR levels peaked between POD #0 and POD #2 across all groups. Postoperative simple main effects analyses demonstrated no statistically significant differences among the three groups at any individual time point from POD #0 to POD #14 (all daily *p* > 0.05).

The LMM analysis demonstrated a significant time effect (Table 3, *p* < 0.001). There was no significant main effect for the hepatic artery size group (group effect, *p* = 0.278) and no significant group-by-time interaction (group × time interaction, *p* = 0.756).

### 3.4. Postoperative Doppler Ultrasonography Findings

Color Doppler ultrasonography was performed daily for the first 7 postoperative days. Quantitative hemodynamic indicators, including peak systolic velocity (PSV), resistive index (RI), and systolic acceleration time (SAT) delay, were analyzed, tracking their longitudinal trajectories.

#### 3.4.1. Hepatic Artery Peak Systolic Velocity (PSV) Alterations

**Figure 4:**
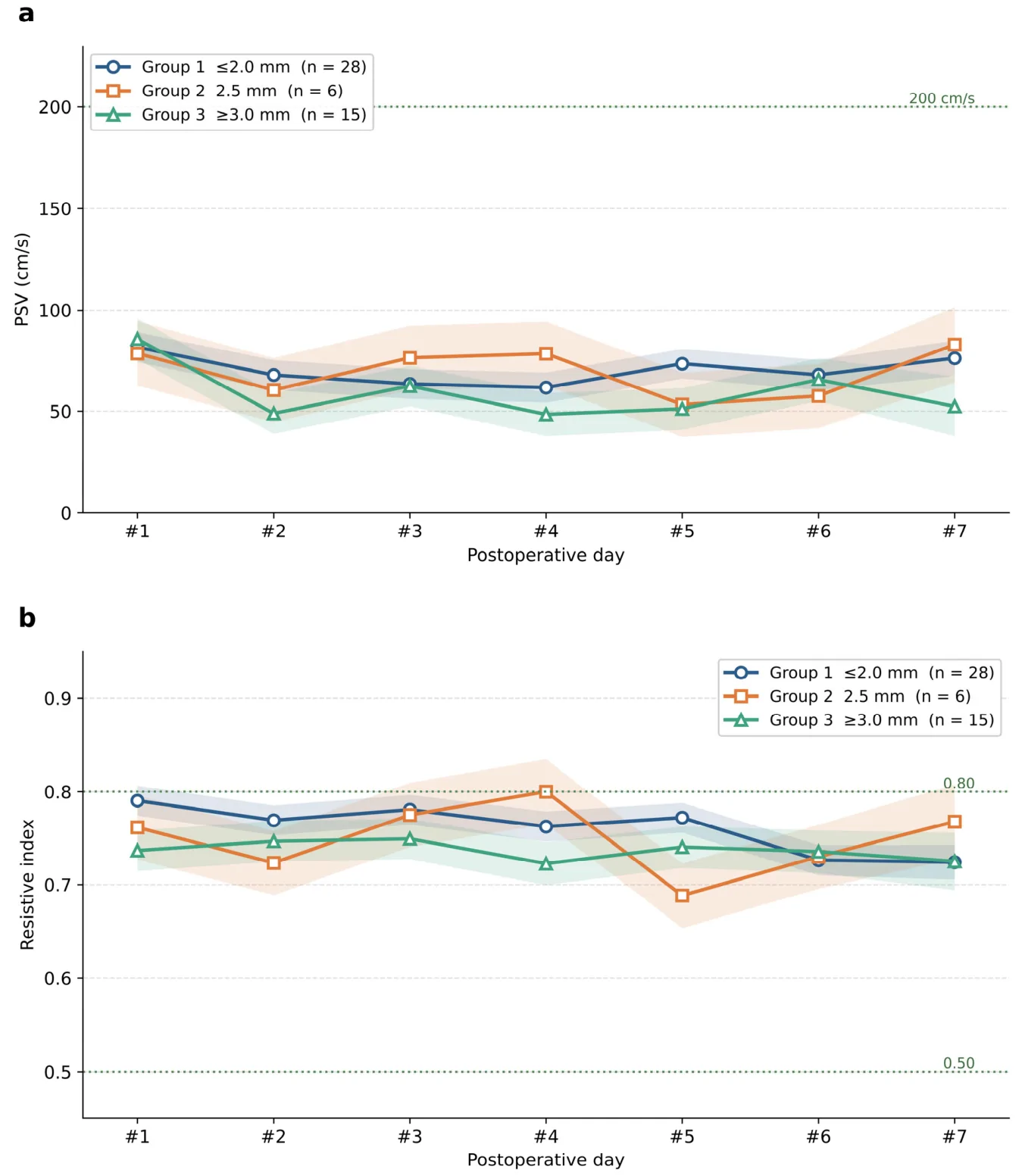
Postoperative hepatic arterial Doppler parameters by hepatic artery (HA) diameter group. (**a**) peak systolic velocity (PSV) and (**b**) resistive index (RI) from postoperative day (POD) 1 through POD 7. Lines represent estimated marginal means from the unadjusted, raw-scale group-based linear mixed models, and shaded areas represent standard errors; *n* is the number of patients in each group (the number of observations per day is given in Supplementary Table S7). Dotted lines indicate reference values for individual examinations, not diagnostic thresholds for group means (see Discussion): (**a**) a PSV of 200 cm/s, above which stenosis is suspected when measured at or just beyond the anastomosis; (**b**) RI values of 0.50 and 0.80, the commonly cited normal range.

The chronological changes in daily hepatic artery PSV values and their corresponding statistical comparisons among the three groups are detailed in Supplementary Table S6 and Figure 4a. From POD #1 to POD #6, the mean PSV values across all three cohorts ranged between 48.4 and 85.7 cm/s. Simple main effects analysis showed no statistically significant differences in PSV among the groups at any single time point during this period (all daily *p* > 0.05). On POD #7, the mean PSV of Group 2 (2.5 mm) was numerically higher (83.0 ± 18.64 cm/s) than that of the other groups (Group 1: 76.2 ± 8.76 cm/s; Group 3: 52.4 ± 14.68 cm/s); however, this difference was not statistically significant (*p* = 0.308).

The comprehensive LMM analysis encompassing the entire 7-day longitudinal dataset showed no significant main effect for the hepatic artery size group (Table 3, *p* = 0.402), no significant temporal variance (*p* = 0.059), and no significant group-by-time interaction (*p* = 0.507).

#### 3.4.2. Hepatic Artery Resistive Index (RI) Alterations

The longitudinal changes in daily hepatic artery RI values and their cross-sectional statistical comparisons are detailed in Supplementary Table S8 and Figure 4b. Throughout the 7-day longitudinal follow-up, the mean daily RI values across all three groups ranged between 0.688 and 0.800.

While overall daily simple main effects analyses showed no statistical significance at any single time point (all overall daily *p* > 0.05), exploratory localized comparisons showed nominal differences at certain time points. On POD #1, the RI in Group 1 (≤2.0 mm) was higher than that in Group 3 (≥3.0 mm) (0.790 ± 0.0160 vs. 0.737 ± 0.0219, unadjusted *p* = 0.049). On POD #5, the RI in Group 1 was higher than that in Group 2 (2.5 mm) (0.772 ± 0.0160 vs. 0.688 ± 0.0346, unadjusted *p* = 0.029). Neither difference remained significant after Holm adjustment. On POD #4, Group 2 reached a numerical peak mean of 0.800 ± 0.035, which subsequently declined to 0.688 ± 0.035 on POD #5. No individual examination recorded an RI below 0.5.

The LMM analysis encompassing the 7-day dataset showed no significant main effect for the hepatic artery size group (Table 3, *p* = 0.466), no significant temporal effect (*p* = 0.107), and no significant interaction between the diameter group and time (*p* = 0.101).

#### 3.4.3. Hepatic Arterial Systolic Acceleration Time (SAT) Delay Analysis

The incidence and chronological distribution of hepatic arterial SAT delay across the three groups are detailed in Table 4. For quantitative analysis, a prolonged systolic upstroke with an SAT of 80 ms or greater was defined as a “delay”.

From POD #1 to #5, patients across all cohorts generally exhibited normal SAT profiles, with no statistically significant differences among the three groups (all *p* > 0.05). On POD #6, the incidence of SAT delay was 11.1% (3/27) in Group 1, 40.0% (2/5) in Group 2, and 0.0% (0/11) in Group 3 (*p* = 0.090). By POD #7, the incidence of SAT delay did not differ significantly among the cohorts (Group 1: 16.7%; Group 2: 25.0%; Group 3: 20.0%; *p* = 1.000). Denominators are the numbers of patients with an SAT measurement on each day (Table 4), and the proportions in Group 2 were based on very small numbers.

### 3.5. Model Diagnostics and Sensitivity Analyses

Residuals of the original models were right-skewed for AST and ALT (skewness 3.35 and 3.94) and moderately skewed for bilirubin, INR and PSV; after log transformation, skewness for AST and ALT decreased to 0.27 and 0.31 (Supplementary Figure S2). On the log scale, neither the group effect (*p* = 0.19–0.70) nor the group × time interaction (*p* = 0.07–0.91) was significant for any outcome.

With HA diameter as a continuous variable, the diameter × time interaction was not significant for any outcome (Table 5). Averaged over time, the estimated effects per 1-mm increase were close to the null for the laboratory outcomes, with wide 95% CIs (e.g., AST ratio 1.01, 95% CI 0.83–1.23; ALT ratio 1.06, 95% CI 0.79–1.42). For PSV, the log-scale ratio was 0.85 (95% CI 0.73–0.98; *p* = 0.030); this was not significant after Holm adjustment across the seven outcomes (*p* = 0.21) and was not observed on the raw scale (*p* = 0.117). Adjustment for GRWR, and additionally for the MELD-Na score, did not materially change any diameter estimate (Table 5). In the GRWR-adjusted models, a higher GRWR was associated with lower total bilirubin (ratio 0.47 per 1-percentage-point increase, 95% CI 0.28–0.79) and lower INR (ratio 0.87, 95% CI 0.78–0.97). In the fully adjusted models, a higher MELD-Na score was associated with higher total bilirubin (ratio 1.05 per point, 95% CI 1.02–1.07) and INR (1.008, 1.003–1.013) and lower ALT (0.95, 0.93–0.97).

After exclusion of the two patients with 4.0- and 5.0-mm arteries, the continuous estimates changed: nominal inverse associations appeared for log total and direct bilirubin (unadjusted *p* = 0.025 and 0.036), whereas the PSV association was no longer present (*p* = 0.22). The continuous results were therefore sensitive to the few patients with the largest diameters.

### 3.6. Early Allograft Dysfunction and Clinical Outcomes

EAD occurred in 5 of 49 patients (10.2%): 3 in Group 1, none in Group 2 and 2 in Group 3 (Table 6). EAD status could not be determined in 3 patients (2 in Group 1 and 1 in Group 3) because POD 7 INR was missing and no other criterion was met; among the 46 patients with a determinable status, EAD occurred in 5 (10.9%). With five events, no formal between-group comparison was performed. One patient in Group 1 underwent splenic artery embolization on POD 6 and POD 16 to reduce portal inflow for small-for-size syndrome and required veno-venous extracorporeal membrane oxygenation for acute respiratory distress syndrome; these were recorded as events unrelated to the HA. Within 90 days, no HAT, HA stenosis requiring intervention, HA re-anastomosis or endovascular treatment, or retransplantation was documented, and no patient died (Table 6). Fourteen patients (28.6%) required biliary intervention within 90 days: 5 of 28 (17.9%) in Group 1, 2 of 6 (33.3%) in Group 2 and 7 of 15 (46.7%) in Group 3 (exploratory Fisher’s exact test, *p* = 0.116). Six patients (12.2%) underwent other reoperations or interventions not involving the HA (5 in Group 1 and 1 in Group 3), including two reoperations for bleeding that did not originate from the HA anastomosis.

## 4. Discussion

In the field of LDLT, a small-caliber HA—particularly one measuring ≤2.0 mm—has traditionally been considered a major risk factor for technical failure, prolonged surgical time, and severe vascular complications such as hepatic artery thrombosis (HAT) [2]. In our analysis of early clinical, biochemical, and hemodynamic outcomes, we found no statistically significant association between donor HA diameter and these outcomes, whether diameter was analyzed in groups or as a continuous variable, and with or without adjustment for GRWR and the MELD-Na score. No arterial complication, retransplantation or death was documented within 90 days. However, the confidence intervals were wide, the study was not designed to test equivalence, and with 49 patients we cannot exclude clinically important differences in uncommon events. Consequently, this study does not show that a small donor HA diameter is safe or that it is not an independent risk factor for graft failure; it indicates that we did not detect statistically significant differences in early surrogate outcomes.

Conventional surgical wisdom suggests that reconstructing small-caliber vessels (≤2.0 mm) is technically more demanding and could consequently prolong the operation and delay recovery [12]. However, our findings demonstrated no statistically significant differences in either total operation time or plastic surgery participation time among the cohorts, although our sample was small and the recorded participation time is not a direct measure of the duration of the arterial anastomosis itself. Furthermore, overall recovery milestones—including the duration of ICU stay, postoperative days until discharge, and total hospital days—did not differ significantly across groups. While not reaching statistical significance, we did observe a trend where larger arterial diameters were associated with heavier liver grafts; however, GRWR did not differ significantly among groups (*p* = 0.403).

In this study, early hepatocellular injury markers (AST and ALT) showed nominal differences during the immediate reperfusion phase (POD #0 and #1), with lower enzyme peaks in the small-caliber cohort (≤2.0 mm) than in the large-diameter group (≥3.0 mm). However, these differences did not remain significant after adjustment for multiple comparisons and were not supported by the continuous-diameter analysis, so they may be chance findings. Although the difference in absolute graft weight among the cohorts did not reach statistical significance (Table 2; *p* = 0.181), we observed a numerical trend where larger arterial diameters were associated with heavier liver grafts. If real, these differences might be partially explained by the proportional relationship between graft volume and absolute hepatocyte mass, which naturally releases a greater total volume of intracellular enzymes upon reperfusion [13]; however, adjustment for GRWR did not change the diameter estimates.

The nominal differences were more pronounced for ALT than for AST. ALT is more liver-specific, whereas AST is also released from skeletal muscle injured during laparotomy [14]; however, because neither difference remained significant after adjustment, we do not draw biological conclusions from this pattern.

Smaller hepatic arteries are considered more susceptible to ischemia–reperfusion injury (IRI) or anastomotic hypoperfusion due to vasospasm [15]. Small-caliber arteries are inherently prone to mechanically induced vasospasm and intimal injury. In our cohort, mean AST levels declined below 100 U/L by POD #4 in all groups, whereas mean ALT levels remained above 100 U/L in all groups through POD #8. This pattern is consistent with, but does not prove, adequate early arterial inflow, because aminotransferase trajectories are influenced by many factors other than arterial caliber.

Following liver transplantation, monitoring of the established criteria for early allograft dysfunction (EAD)—specifically AST, ALT, bilirubin, and INR—is important for predicting short-term graft survival [11,16]. Among these parameters, prolonged elevation in serum bilirubin is widely recognized in the literature as a critical predictor of EAD and subsequent graft failure [17]. In the present study, both total and direct bilirubin, alongside INR, showed no significant dependency on the intraoperative HA diameter. Patient-level EAD by the Olthoff definition occurred in 5 of 49 patients, and its status was indeterminate in 3; with so few events, a comparison between groups was not informative, and group-mean trajectories cannot replace patient-level assessment.

This point is relevant when considering the specific anatomical blood supply of the liver graft. Unlike the hepatic parenchyma, which receives dual perfusion from both the portal vein and the hepatic artery, the biliary epithelium relies almost entirely on the arterial blood supply via the peribiliary capillary plexus [18]. Consequently, hemodynamically significant hypoperfusion in a small-caliber HA (≤2.0 mm) could manifest as ischemic cholangiopathy [19]. We found no significant group differences in direct bilirubin or INR trajectories up to POD 14, and biliary intervention within 90 days was not more frequent in Group 1 (17.9%, compared with 33.3% in Group 2 and 46.7% in Group 3; *p* = 0.116). However, the cause of these biliary complications was not classified, and later ischemic complications beyond 90 days were not captured, so these data do not establish adequate arterial oxygenation of the biliary tree.

Daily Doppler ultrasonography provided additional information on the microvascular anastomoses. Among the hemodynamic parameters, PSV is a primary indicator of arterial inflow and structural patency. A PSV above 200 cm/s at or just beyond the anastomosis suggests stenosis, whereas a low PSV with a tardus parvus waveform distal to the anastomosis raises concern for impaired inflow; however, a wide range of PSV values has been reported in the early postoperative period [20]. Throughout the 7-day longitudinal follow-up, the mean daily PSV in each group remained below 200 cm/s (range of group means, 48.4–85.7 cm/s). In the continuous log-scale model, PSV tended to be lower with larger diameters, but this finding did not withstand multiplicity adjustment and was not robust in sensitivity analyses. Group means within a reference range do not exclude abnormal values in individual patients.

Regarding the RI, which reflects downstream microvascular resistance, the normal physiological range during the early post-transplant phase is approximately 0.50 to 0.80, although transient values above 0.80 are common in the first days after transplantation [20,21]. In our results, although exploratory comparisons showed nominally higher RI values in the small-caliber cohort (≤2.0 mm) at certain time points (POD #1 and POD #5), these differences did not remain significant after multiplicity adjustment, and the mean RI values across all groups remained within the normal physiological margin (0.688–0.800). No individual examination recorded an RI below 0.5. Transient elevation in RI may reflect a physiological adaptation—such as mild vasospasm or graft edema—rather than pathological congestion [22].

Finally, analyzing the SAT provided information on proximal arterial inflow. A tardus-parvus waveform, conventionally defined by a prolonged SAT (>80 ms) together with a low RI (<0.5), suggests significant upstream stenosis [23,24]. In this study, an SAT of ≥80 ms alone was used as an operational definition of delayed systolic upstroke and does not by itself indicate stenosis. After correction of the denominators, no daily between-group difference in SAT delay was statistically significant, although the proportion of patients with delay was numerically higher in Group 1 than in Group 3 on six of the seven days. The small number of patients in Group 2 precludes meaningful interpretation of its proportions. Because no examination recorded an RI below 0.5, no examination showed SAT prolongation combined with a low RI.

All arterial anastomoses in this cohort were performed through collaboration between the General Surgery (GS) and Plastic Surgery (PS) teams at our institution. While the GS team focuses on macro-surgical components—such as graft procurement, bench surgery, and recipient hepatectomy—the PS team specializes in high-magnification microvascular surgery [3]. Shifting the technical burden of delicate micro-anastomosis to dedicated microsurgeons may help achieve precise intimal apposition and flatten the learning curve typically associated with small-vessel reconstructions [25]. However, because there was no comparison group without microsurgical involvement, our data cannot show whether this collaboration reduced any technical disadvantage of small-caliber arteries.

This study has several limitations. First, the retrospective, single-center design inherently limits our ability to establish causality, and our findings may reflect the specific surgical practices of our institutional transplantation team. Second, the sample size—particularly in the 2.5 mm cohort (*n* = 6)—was small, which limits precision; the study was not designed to test equivalence, and larger, multi-center validation remains necessary. Third, the diameter categories were defined after data collection according to the 0.5-mm recording resolution rather than an established biological threshold, and the continuous analyses, added post hoc, were sensitive to the two patients with the largest diameters. Fourth, diameter was measured by visual comparison with a gauge at 0.5-mm resolution, and vasoconstriction from cold perfusion, handling, topical papaverine timing or systemic vasopressors could not be accounted for; such measurement error would tend to attenuate associations.

Fifth, residual confounding is likely. We adjusted only for GRWR and the MELD-Na score, which was calculated after preoperative plasmapheresis in two patients; donor age and sex, graft steatosis, cold and warm ischemia times, blood loss and transfusion, portal flow, vasopressor use, recipient HA diameter (and therefore donor–recipient arterial size mismatch), the recipient inflow artery, gastroduodenal artery ligation and arterial length were not available in a consistent form. Sixth, the primary outcomes were biochemical and Doppler surrogates; the study was underpowered for clinically important but uncommon arterial events, asymptomatic HA stenosis was not systematically screened for, and the causes of biliary complications were not classified. Seventh, observations decreased after the first postoperative week, reasons for missing data were not recorded, and missingness may be related to the clinical course, so the missing-at-random assumption of the mixed models may not fully hold. Finally, our study does not extend to late-stage complications such as chronic rejection or long-term biliary strictures, and future prospective studies with extended follow-up are required.

## 5. Conclusions

In conclusion, in this single-center cohort of 49 LDLT recipients, we found no evidence that an intraoperative hepatic artery diameter of ≤2.0 mm was associated with worse early biochemical or Doppler trajectories than larger diameters, in either group-based or continuous analyses, and no arterial complication was documented within 90 days. The estimates were imprecise, the longitudinal analyses relied on biochemical and Doppler surrogates, the study was underpowered for uncommon clinical events, and adjustment was limited to the available covariates; these findings therefore do not establish equivalence or vascular safety. Larger studies with adjusted analyses and clinically defined arterial and biliary outcomes are needed to determine whether donor HA diameter should influence surgical planning or donor selection.

## Figures and Tables

**Table 1:** Baseline characteristics of study participants by hepatic artery diameter group. Data are presented as mean ± SD for continuous variables and *n* for categorical variables. HCC: hepatocellular carcinoma; HBV: hepatitis B virus; HCV: hepatitis C virus; SD: standard deviation.

Variable	≤2 mm (*N* = 28)	2.5 mm (*N* = 6)	≥3 mm (*N* = 15)	Total (*N* = 49)
Total number (*N*)	28	6	15	49
Sex				
Male	21	4	11	36
Female	7	2	4	13
Age (Mean ± SD)	55.9 ± 10.8	58.0 ± 8.9	58.8 ± 11.0	57.1 ± 10.6
Diagnosis				
Alcoholic (only)	14	1	6	21
HCC (only)	8	2	6	16
Combined (Alcoholic + HCC)	5	2	0	7
Others	1	1	3	5
Virus				
HBV	6	2	4	12
HCV	2	0	0	2

**Table 2:** Intraoperative and postoperative clinical parameters by hepatic artery diameter group. Comparisons were performed using Welch’s one-way ANOVA or, where indicated (†), the Kruskal–Wallis test. Data are presented as mean ± SD, median (IQR) or *n* (%). PS participation time: time of plastic surgery team participation as recorded in the operative records, including bench work. GRWR: graft-to-recipient weight ratio; MELD-Na: Model for End-Stage Liver Disease-sodium score; IQR: interquartile range. † Kruskal–Wallis test. HD: hospital day; POD: postoperative day; ICU: intensive care unit; SD: standard deviation.

Variable	≤2 mm (*n* = 28)	2.5 mm (*n* = 6)	≥3 mm (*n* = 15)	*p*-Value
HD (days)	52.18 ± 61.38	35.00 ± 28.04	34.87 ± 18.92	0.419
POD (days)	33.50 ± 38.08	29.17 ± 20.22	25.47 ± 8.34	0.562
ICU time (days)	8.89 ± 6.59	6.67 ± 1.51	6.13 ± 1.06	0.123
Operation time (min)	626.75 ± 86.51	552.67 ± 81.33	629.07 ± 123.02	0.170
PS participation time (min)	93.93 ± 52.01	70.67 ± 31.21	87.20 ± 53.37	0.384
Liver weight (g)	683.84 ± 137.62	755.83 ± 203.80	770.80 ± 137.33	0.181
Recipient weight (kg)	63.29 ± 12.71	68.48 ± 11.80	63.65 ± 16.07	0.644
GRWR (%)	1.12 ± 0.32	1.11 ± 0.26	1.28 ± 0.39	0.403
GRWR < 0.8%, *n* (%)	5 (17.9)	1 (16.7)	1 (6.7)	–
Right-lobe graft, *n* (%)	28 (100)	6 (100)	15 (100)	–
MELD-Na score, median (IQR)	14.0 (8.8–18.2)	10.5 (10.0–13.2)	11.0 (9.5–15.0)	0.727 †

**Table 3:** Fixed effects omnibus tests from the linear mixed models for all outcome variables. The main effects of hepatic artery diameter group and time (date), as well as their interaction effect (Group × Date), were evaluated as fixed effects within the model framework. AST: aspartate aminotransferase; ALT: alanine aminotransferase; INR: international normalized ratio; PSV: peak systolic velocity; RI: resistive index. * *p* < 0.05.

Outcome	Effect	*p*-Value
Serum Laboratory Tests		
AST	Group	0.758
	Date	<0.001 *
	Group × Date	0.995
ALT	Group	0.837
	Date	<0.001 *
	Group × Date	0.708
Total Bilirubin	Group	0.547
	Date	<0.001 *
	Group × Date	0.923
Direct Bilirubin	Group	0.574
	Date	0.172
	Group × Date	0.948
INR	Group	0.278
	Date	<0.001 *
	Group × Date	0.756
Doppler Ultrasonography		
PSV	Group	0.402
	Date	0.059
	Group × Date	0.507
RI	Group	0.466
	Date	0.107
	Group × Date	0.101

**Table 4:** Incidence of hepatic artery systolic acceleration time (SAT) delay by hepatic artery diameter group. A prolonged systolic upstroke with SAT ≥ 80 ms was defined as delay. Daily comparisons used Fisher’s exact test without adjustment for multiplicity. Data are *n*/*N* (%), where *N* is the number of patients with SAT measured on that day; group sizes in the header are the numbers of patients in each group. SAT: systolic acceleration time; POD: postoperative day.

	Status	≤2 mm (*n* = 28)	2.5 mm (*n* = 6)	≥3 mm (*n* = 15)	*p*-Value
POD #1	delay	2/28 (7.1%)	0/5 (0.0%)	0/12 (0.0%)	1.000
	normal	26/28 (92.9%)	5/5 (100.0%)	12/12 (100.0%)	
POD #2	delay	6/28 (21.4%)	0/5 (0.0%)	0/13 (0.0%)	0.159
	normal	22/28 (78.6%)	5/5 (100.0%)	13/13 (100.0%)	
POD #3	delay	6/28 (21.4%)	0/5 (0.0%)	1/13 (7.7%)	0.479
	normal	22/28 (78.6%)	5/5 (100.0%)	12/13 (92.3%)	
POD #4	delay	2/27 (7.4%)	0/5 (0.0%)	0/11 (0.0%)	1.000
	normal	25/27 (92.6%)	5/5 (100.0%)	11/11 (100.0%)	
POD #5	delay	4/28 (14.3%)	2/5 (40.0%)	0/12 (0.0%)	0.081
	normal	24/28 (85.7%)	3/5 (60.0%)	12/12 (100.0%)	
POD #6	delay	3/27 (11.1%)	2/5 (40.0%)	0/11 (0.0%)	0.090
	normal	24/27 (88.9%)	3/5 (60.0%)	11/11 (100.0%)	
POD #7	delay	3/18 (16.7%)	1/4 (25.0%)	1/5 (20.0%)	1.000
	normal	15/18 (83.3%)	3/4 (75.0%)	4/5 (80.0%)	

**Table 5:** Association between continuous HA diameter and postoperative outcomes (linear mixed models, log-transformed outcomes). Ratios are per 1-mm increase in HA diameter, averaged over time points, from the model including the HA diameter × time interaction. Raw-scale estimates are given in Supplementary Table S9. CI: confidence interval; GRWR: graft-to-recipient weight ratio; MELD-Na: Model for End-Stage Liver Disease-sodium score; PSV: peak systolic velocity; RI: resistive index.

Outcome	Ratio per 1 mm (95% CI)	*p* (Diameter)	*p* (Diameter × Time)	Adjusted Ratio (95% CI) †
AST	1.01 (0.83–1.23)	0.909	0.879	1.02 (0.84–1.23)
ALT	1.06 (0.79–1.42)	0.703	0.669	1.07 (0.84–1.36)
Total bilirubin	0.94 (0.69–1.28)	0.690	0.987	0.94 (0.73–1.20)
Direct bilirubin	0.88 (0.60–1.28)	0.487	0.987	0.87 (0.64–1.18)
INR	0.97 (0.91–1.04)	0.393	0.841	0.97 (0.92–1.03)
PSV	0.85 (0.73–0.98)	0.030 *	0.205	0.85 (0.73–0.99)
RI	0.98 (0.94–1.02)	0.218	0.553	0.98 (0.94–1.01)

* Not significant after Holm adjustment across the seven outcomes (adjusted *p* = 0.21). † Adjusted for GRWR and the preoperative MELD-Na score. Observations (patients): 731 (49) for AST, ALT and bilirubin; 696 (49) for INR; 314 (49) for PSV; 315 (49) for RI.

**Table 6:** Patient-level early allograft dysfunction and clinical outcomes within 90 days by hepatic artery diameter group. Data are *n* (%). EAD: early allograft dysfunction (Olthoff definition); HAT: hepatic artery thrombosis; HA: hepatic artery. Biliary intervention: percutaneous biloma drainage, percutaneous transhepatic biliary drainage, endoscopic retrograde cholangiopancreatography or surgical repair; the cause (anastomotic or ischemic) was not classified. Other: reoperation for bleeding (*n* = 2), hernia repair with small-bowel resection, abscess drainage, wound dehiscence repair, and splenic artery embolization for portal inflow modulation.

Outcome	≤2 mm (*n* = 28)	2.5 mm (*n* = 6)	≥3 mm (*n* = 15)	Total (*n* = 49)
EAD	3 (10.7)	0 (0.0)	2 (13.3)	5 (10.2)
EAD indeterminate	2 (7.1)	0 (0.0)	1 (6.7)	3 (6.1)
HAT	0	0	0	0
HA stenosis requiring intervention	0	0	0	0
HA re-anastomosis or endovascular treatment	0	0	0	0
Retransplantation	0	0	0	0
Biliary intervention	5 (17.9)	2 (33.3)	7 (46.7)	14 (28.6)
Other reoperation or non-HA intervention	5 (17.9)	0 (0.0)	1 (6.7)	6 (12.2)
30-day mortality	0	0	0	0
90-day mortality	0	0	0	0

## Data Availability

The data presented in this study are available on request from the corresponding author due to the data contain clinical information subject to patient-confidentiality restrictions.

## Supplementary Materials

The following supporting information can be downloaded at: https://www.mdpi.com/article/10.3390/medicina62102006/s1. Table S1: Postoperative serum aspartate aminotransferase (AST) levels by hepatic artery diameter group; Table S2: Postoperative serum alanine aminotransferase (ALT) levels by hepatic artery diameter group; Table S3: Postoperative serum total bilirubin levels by hepatic artery diameter group; Table S4: Postoperative serum direct bilirubin levels by hepatic artery diameter group; Table S5: Postoperative serum international normalized ratio (INR) levels by hepatic artery diameter group; Table S6: Postoperative hepatic artery peak systolic velocity (PSV) by hepatic artery diameter group; Table S7: Number of patients with an observation at each time point (*n* = 49); Table S8: Postoperative hepatic artery resistive index (RI) by hepatic artery diameter group; Table S9: Association between continuous hepatic artery diameter and postoperative outcomes on the raw scale; Figure S1: Postoperative serum direct bilirubin levels by hepatic artery diameter group; Figure S2: Residual diagnostics of the group × time linear mixed models.
