# Supplementary material for: Impact of Hepatic Artery Diameter on Immediate Postoperative Graft Function and Hemodynamic Profiles in Living-Donor Liver Transplantation: A Retrospective Cohort Study
Source: Medicina (Kaunas). 2026 Oct 16;62(10):2006. doi: 10.3390/medicina62102006 (PMC13706735; doi:10.3390/medicina62102006)

Supplementary Information

Impact of Hepatic Artery Diameter on Immediate Postoperative Graft Function and Hemodynamic Profiles in Living Donor Liver Transplantation: A Retrospective Cohort Study

Il Young Ahn et al.

Supplementary Table S1. Postoperative serum aspartate aminotransferase (AST) levels by hepatic artery diameter group.

**AST**	**≤2mm (*n*=28)**	**2.5mm (*n*=6)**	**≥3mm (*n*=15)**	***P*-value**
Pre-operative	44.2 ± 27.4	48.2 ± 59.1	53.3 ± 37.4	0.981
POD #0	359.9 ± 27.4	402.0 ± 59.1	461.5 ± 37.4	0.091
POD #1	395.9 ± 27.4	498.2 ± 59.1	497.5 ± 37.4	0.052
POD #2	262.4 ± 27.4	273.0 ± 59.1	278.5 ± 37.4	0.938
POD #3	130.7 ± 27.4	131.5 ± 59.1	139.9 ± 37.4	0.98
POD #4	75.3 ± 27.4	85.3 ± 59.1	71.6 ± 37.4	0.981
POD #5	71.9 ± 27.4	70.8 ± 59.1	49.4 ± 37.4	0.884
POD #6	78.9 ± 27.4	72.7 ± 59.1	52.5 ± 37.4	0.849
POD #7	62.0 ± 27.4	70.7 ± 59.1	50.5 ± 37.4	0.950
POD #8	56.7 ± 27.8	71.7 ± 59.1	59.7 ± 37.4	0.974
POD #9	52.1 ± 28.3	82.3 ± 64.0	56.7 ± 37.4	0.911
POD #10	62.7 ± 27.4	78.8 ± 59.1	54.1 ± 38.5	0.940
POD #11	59.5 ± 32.5	93.0 ± 80.7	34.3 ± 42.8	0.790
POD #12	57.7 ± 29.3	78.8 ± 59.1	57.8 ± 39.8	0.946
POD #13	46.7 ± 31.0	88.1 ± 70.7	42.2 ± 42.8	0.846
POD #14	48.3 ± 31.0	66.5 ± 59.1	72.3 ± 46.9	0.900

AST, aspartate aminotransferase; POD, postoperative day; SE, standard error. Values are expressed as mean ± SE (linear mixed model). *P* values are from tests of the simple main effect of group at each time point and are not adjusted for multiple comparisons; these time-point-specific comparisons are exploratory. * Unadjusted $P < 0.05$.

Supplementary Table S2. Postoperative serum alanine aminotransferase (ALT) levels by hepatic artery diameter group.

ALT	≤2mm (*n*=28)	2.5mm (*n*=6)	≥3mm (*n*=15)	*P*-value
Pre-operative	25.9 ± 44.9	18.2 ± 97.0	27.9 ± 61.3	0.996
POD #0	292.4 ± 44.9	325.5 ± 97.0	526.5 ± 61.3	0.009*
POD #1	398.4 ± 44.9	468.0 ± 97.0	588.4 ± 61.3	0.046*
POD #2	410.5 ± 44.9	398.3 ± 97.0	470.5 ± 61.3	0.697
POD #3	313.4 ± 44.9	264.5 ± 97.0	348.3 ± 61.3	0.756
POD #4	221.9 ± 44.9	194.3 ± 97.0	258.3 ± 61.3	0.827
POD #5	183.6 ± 44.9	164.5 ± 97.0	188.1 ± 61.3	0.979
POD #6	161.5 ± 44.9	154.7 ± 97.0	147.6 ± 61.3	0.983
POD #7	143.0 ± 44.9	138.8 ± 97.0	120.9 ± 61.3	0.958
POD #8	126.0 ± 45.5	126.8 ± 97.0	112.7 ± 61.3	0.984
POD #9	99.6 ± 46.1	135.2 ± 103.4	102.0 ± 61.3	0.951
POD #10	115.9 ± 44.9	132.8 ± 97.0	97.6 ± 62.8	0.949
POD #11	102.0 ± 51.7	137.1 ± 125.9	58.8 ± 68.6	0.819
POD #12	107.0 ± 47.4	133.3 ± 97.0	98.8 ± 64.5	0.956
POD #13	99.0 ± 49.8	150.2 ± 112.4	77.5 ± 68.6	0.859
POD #14	96.6 ± 49.8	121.8 ± 97.0	132.0 ± 74.0	0.916

ALT, alanine aminotransferase; POD, postoperative day; SE, standard error. Values are expressed as mean ± SE (linear mixed model). *P* values are from tests of the simple main effect of group at each time point and are not adjusted for multiple comparisons; these time-point-specific comparisons are exploratory. * Unadjusted $P < 0.05$.

Supplementary Table S3. Postoperative serum total bilirubin levels by hepatic artery diameter group.

**Total Bilirubin**	**≤2mm (*n*=28)**	**2.5mm (*n*=6)**	**≥3mm (*n*=15)**	***P*-value**
Pre-operative	2.750 ± 0.492	3.150 ± 1.062	2.660 ± 0.672	0.925
POD #0	4.218 ± 0.492	4.483 ± 1.062	3.887 ± 0.672	0.873
POD #1	4.571 ± 0.492	4.450 ± 1.062	3.740 ± 0.672	0.602
POD #2	2.668 ± 0.492	2.900 ± 1.062	2.380 ± 0.672	0.902
POD #3	2.186 ± 0.492	2.000 ± 1.062	2.107 ± 0.672	0.986
POD #4	2.350 ± 0.492	1.850 ± 1.062	2.240 ± 0.672	0.913
POD #5	2.682 ± 0.492	1.800 ± 1.062	2.233 ± 0.672	0.705
POD #6	2.671 ± 0.492	1.633 ± 1.062	2.293 ± 0.672	0.656
POD #7	2.668 ± 0.492	1.383 ± 1.062	2.347 ± 0.672	0.546
POD #8	2.676 ± 0.496	1.283 ± 1.062	2.247 ± 0.672	0.484
POD #9	2.517 ± 0.500	1.052 ± 1.106	2.020 ± 0.672	0.463
POD #10	2.504 ± 0.492	1.067 ± 1.062	2.000 ± 0.682	0.452
POD #11	2.993 ± 0.538	0.712 ± 1.264	1.836 ± 0.721	0.168
POD #12	2.469 ± 0.508	0.800 ± 1.062	1.527 ± 0.693	0.278
POD #13	2.386 ± 0.525	0.883 ± 1.168	1.532 ± 0.721	0.396
POD #14	2.385 ± 0.525	0.833 ± 1.062	1.520 ± 0.759	0.352

POD, postoperative day; SE, standard error. Values are expressed as mean ± SE (linear mixed model). *P* values are from tests of the simple main effect of group at each time point and are not adjusted for multiple comparisons; these time-point-specific comparisons are exploratory.
* Unadjusted $P < 0.05$.

Supplementary Table S4. Postoperative serum direct bilirubin levels by hepatic artery diameter group.

Direct Bilirubin	≤2mm (*n*=28)	2.5mm (*n*=6)	≥3mm (*n*=15)	*P*-value
Pre-operative	1.236 ± 0.367	1.533 ± 0.794	1.633 ± 0.502	0.801
POD #0	1.879 ± 0.367	1.750 ± 0.794	1.753 ± 0.502	0.975
POD #1	2.089 ± 0.367	1.733 ± 0.794	1.653 ± 0.502	0.760
POD #2	1.339 ± 0.367	1.367 ± 0.794	1.067 ± 0.502	0.899
POD #3	1.257 ± 0.367	1.017 ± 0.794	1.187 ± 0.502	0.962
POD #4	1.439 ± 0.367	1.033 ± 0.794	1.360 ± 0.502	0.898
POD #5	1.682 ± 0.367	1.000 ± 0.794	1.360 ± 0.502	0.698
POD #6	1.679 ± 0.367	0.867 ± 0.794	1.393 ± 0.502	0.633
POD #7	1.754 ± 0.367	0.733 ± 0.794	1.480 ± 0.502	0.503
POD #8	1.785 ± 0.370	0.717 ± 0.794	1.453 ± 0.502	0.465
POD #9	1.679 ± 0.372	0.624 ± 0.822	1.340 ± 0.502	0.490
POD #10	1.675 ± 0.367	0.550 ± 0.794	1.374 ± 0.508	0.434
POD #11	1.965 ± 0.398	0.432 ± 0.927	1.235 ± 0.534	0.240
POD #12	1.659 ± 0.378	0.417 ± 0.794	1.059 ± 0.516	0.312
POD #13	1.556 ± 0.389	0.489 ± 0.863	1.029 ± 0.534	0.460
POD #14	1.571 ± 0.389	0.400 ± 0.794	1.053 ± 0.559	0.381

POD, postoperative day; SE, standard error. Values are expressed as mean ± SE (linear mixed model). *P* values are from tests of the simple main effect of group at each time point and are not adjusted for multiple comparisons; these time-point-specific comparisons are exploratory.
* Unadjusted $P < 0.05$.

Supplementary Table S5. Postoperative serum international normalized ratio (INR) levels by hepatic artery diameter group.

**INR**	**≤2mm (*n*=28)**	**2.5mm (*n*=6)**	**≥3mm (*n*=15)**	***P*-value**
Pre-operative	1.452 ± 0.0551	1.613 ± 0.1190	1.292 ± 0.0753	0.055
POD #0	1.682 ± 0.0551	1.613 ± 0.1190	1.739 ± 0.0753	0.654
POD #1	1.767 ± 0.0551	1.750 ± 0.1190	1.683 ± 0.0753	0.659
POD #2	1.721 ± 0.0551	1.475 ± 0.1190	1.540 ± 0.0753	0.056
POD #3	1.354 ± 0.0551	1.197 ± 0.1190	1.247 ± 0.0753	0.333
POD #4	1.265 ± 0.0551	1.132 ± 0.1190	1.176 ± 0.0753	0.458
POD #5	1.230 ± 0.0551	1.188 ± 0.1190	1.205 ± 0.0753	0.932
POD #6	1.250 ± 0.0551	1.147 ± 0.1190	1.225 ± 0.0770	0.732
POD #7	1.285 ± 0.0565	1.098 ± 0.1190	1.196 ± 0.0790	0.311
POD #8	1.245 ± 0.0572	1.093 ± 0.1190	1.157 ± 0.0811	0.428
POD #9	1.231 ± 0.0572	1.045 ± 0.1263	1.210 ± 0.0790	0.405
POD #10	1.180 ± 0.0565	1.060 ± 0.1190	1.111 ± 0.0836	0.587
POD #11	1.142 ± 0.0654	0.943 ± 0.1519	1.033 ± 0.0899	0.376
POD #12	1.132 ± 0.0597	1.043 ± 0.1190	0.997 ± 0.0836	0.401
POD #13	1.073 ± 0.0654	0.995 ± 0.1365	1.007 ± 0.0899	0.784
POD #14	1.127 ± 0.0628	1.057 ± 0.1190	0.959 ± 0.0939	0.329

INR, international normalized ratio; POD, postoperative day; SE, standard error. Values are expressed as mean ± SE (linear mixed model). *P* values are from tests of the simple main effect of group at each time point and are not adjusted for multiple comparisons; these time-point-specific comparisons are exploratory. * Unadjusted $P < 0.05$.

Supplementary Table S6. Postoperative hepatic artery peak systolic velocity (PSV) by hepatic artery diameter group.

PSV	≤2mm (*n*=28)	2.5mm (*n*=6)	≥3mm (*n*=15)	*P*-value
POD #1	81.7 ± 7.45	78.6 ± 15.88	85.7 ± 10.05	0.917
POD #2	67.9 ± 7.35	60.4 ± 15.88	48.9 ± 10.05	0.313
POD #3	63.5 ± 7.35	76.4 ± 15.88	62.5 ± 10.05	0.734
POD #4	61.7 ± 7.46	78.5 ± 15.88	48.4 ± 10.62	0.275
POD #5	73.5 ± 7.35	53.3 ± 15.88	51.1 ± 10.32	0.163
POD #6	68.0 ± 7.45	57.5 ± 15.88	65.6 ± 10.62	0.837
POD #7	76.2 ± 8.76	83.0 ± 18.64	52.4 ± 14.68	0.308

PSV, peak systolic velocity; POD, postoperative day; SE, standard error. Values are expressed as mean ± SE (linear mixed model). *P* values are from tests of the simple main effect of group at each time point and are not adjusted for multiple comparisons; these time-point-specific comparisons are exploratory. * Unadjusted $P < 0.05$.

Supplementary Table S7. Number of patients with an observation at each time point (*n* = 49)

Number of patients with an observation at each time point (*n* = 49). Reasons for missing measurements were not systematically recorded.

Outcome	PRE	POD0	POD1	POD2	POD3	POD4	POD5	POD6	POD7	POD8	POD9	POD10	POD11	POD12	POD13	POD14	Total
AST	49	49	49	49	49	49	49	49	49	48	46	48	33	43	36	36	731
ALT	49	49	49	49	49	49	49	49	49	48	46	48	33	43	36	36	731
Total bilirubin	49	49	49	49	49	49	49	49	49	48	46	48	33	43	36	36	731
Direct bilirubin	49	49	49	49	49	49	49	49	49	48	46	48	33	43	36	36	731
INR	49	49	49	49	49	49	49	48	45	43	43	43	29	39	30	33	696

PRE, preoperative; POD, postoperative day.

Outcome	POD1	POD2	POD3	POD4	POD5	POD6	POD7	Total
PSV	48	49	49	46	48	46	28	314
RI	49	49	49	46	48	46	28	315

PSV, peak systolic velocity; RI, resistive index.

Supplementary Figure S1. Postoperative serum direct bilirubin levels by hepatic artery diameter group

Postoperative serum direct bilirubin levels by hepatic artery diameter group from the preoperative period through postoperative day (POD) 14. Lines represent estimated means and shaded areas represent standard errors.

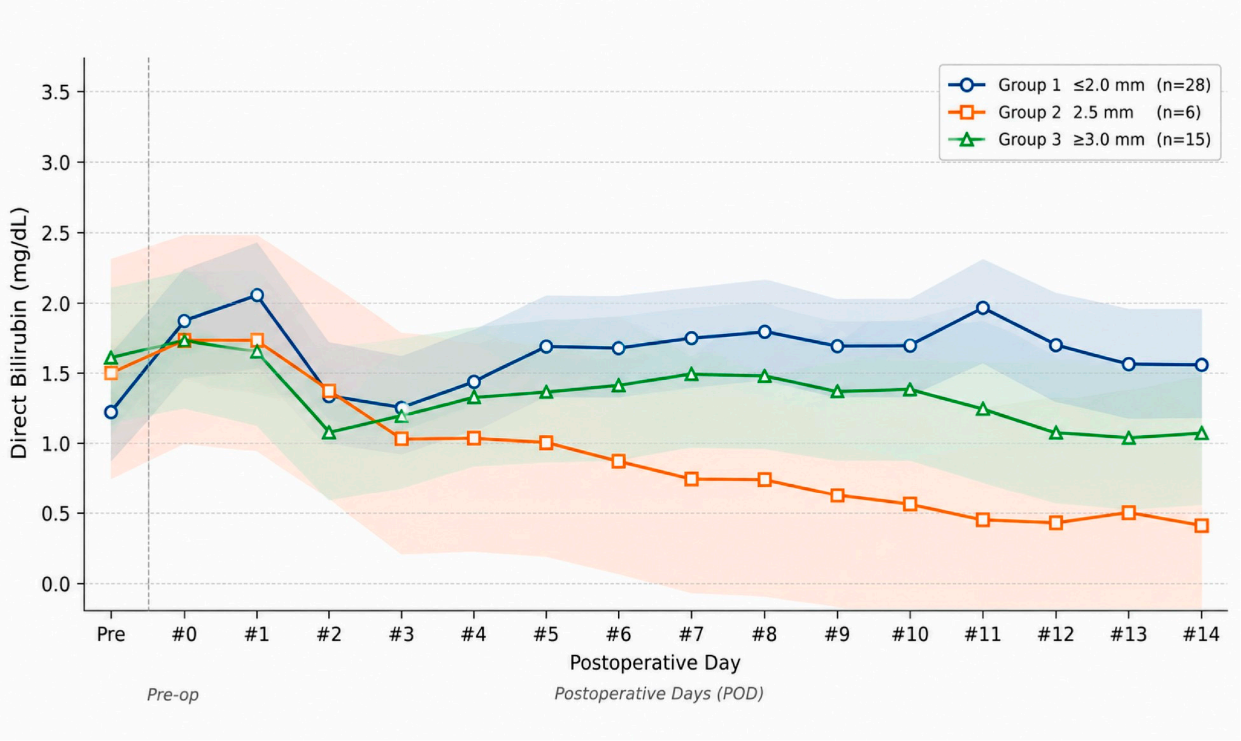

Supplementary Table S8. Postoperative hepatic artery resistive index (RI) by hepatic artery diameter group.

RI	≤2mm (*n*=28)	2.5mm (*n*=6)	≥3mm (*n*=15)	*P*-value
POD #1	0.790 ± 0.0160	0.762 ± 0.0346	0.737 ± 0.0219	0.139
POD #2	0.769 ± 0.0160	0.723 ± 0.0346	0.747 ± 0.0219	0.417
POD #3	0.781 ± 0.0160	0.775 ± 0.0346	0.749 ± 0.0219	0.508
POD #4	0.763 ± 0.0162	0.800 ± 0.0346	0.723 ± 0.0230	0.146
POD #5	0.772 ± 0.0160	0.688 ± 0.0346	0.740 ± 0.0224	0.076
POD #6	0.726 ± 0.0162	0.730 ± 0.0346	0.735 ± 0.0230	0.948
POD #7	0.724 ± 0.0187	0.768 ± 0.0399	0.725 ± 0.0309	0.601

RI, resistive index; POD, postoperative day; SE, standard error. Values are expressed as mean ± SE (linear mixed model). *P* values are from tests of the simple main effect of group at each time point and are not adjusted for multiple comparisons; these time-point-specific comparisons are exploratory. * Unadjusted $P < 0.05$.

Supplementary Table S9. Association between continuous hepatic artery diameter and postoperative outcomes on the raw scale

Association between continuous hepatic artery diameter and postoperative outcomes on the raw (untransformed) scale. B: difference in the outcome per 1-mm increase in HA diameter, averaged over time points, from the model with the HA diameter × time interaction; common-slope B from the model without interaction.

Outcome (unit)	B per 1 mm (95% CI)	*p* (diameter)	*p* (diameter × time)	Adjusted B (95% CI) †	Common-slope B (95% CI)
AST (U/L)	3.3 (−29.6 to 36.1)	0.843	0.996	4.4 (−27.8 to 36.5)	3.9 (−28.7 to 36.5)
ALT (U/L)	8.3 (−61.3 to 78.0)	0.811	0.785	10.8 (−53.3 to 75.0)	11.0 (−58.5 to 80.5)
Total bilirubin (mg/dL)	0.01 (−0.96 to 0.98)	0.983	0.927	−0.01 (−0.82 to 0.81)	0.05 (−0.92 to 1.02)
Direct bilirubin (mg/dL)	0.03 (−0.73 to 0.78)	0.943	0.600	0.01 (−0.64 to 0.66)	0.05 (−0.70 to 0.80)
INR	−0.036 (−0.128 to 0.057)	0.443	0.922	−0.037 (−0.115 to 0.041)	−0.032 (−0.125 to 0.060)
PSV (cm/s)	−9.7 (−22.0 to 2.5)	0.117	0.574	−10.0 (−22.4 to 2.4)	−8.4 (−20.3 to 3.6)
RI	−0.019 (−0.048 to 0.009)	0.185	0.626	−0.020 (−0.048 to 0.009)	−0.021 (−0.049 to 0.008)

Linear mixed models with a random intercept per patient, REML, Satterthwaite degrees of freedom. † Adjusted for GRWR and the preoperative MELD-Na score (log-scale counterparts in Table 5 of the main text).
GRWR, graft-to-recipient weight ratio; MELD-Na, Model for End-Stage Liver Disease-sodium score; CI, confidence interval; PSV, peak systolic velocity; RI, resistive index.

Supplementary Figure S2. Residual diagnostics of the group × time linear mixed models.

Residual diagnostics of the group × time linear mixed models for each outcome, on the raw scale (rows 1–2) and after natural-log transformation (rows 3–4). Rows 1 and 3: normal Q–Q plots of conditional residuals (observed values minus fitted values including the patient random intercept), with residual skewness shown in each panel. Rows 2 and 4: conditional residuals versus fitted values. PSV, peak systolic velocity; RI, resistive index; INR, international normalized ratio.

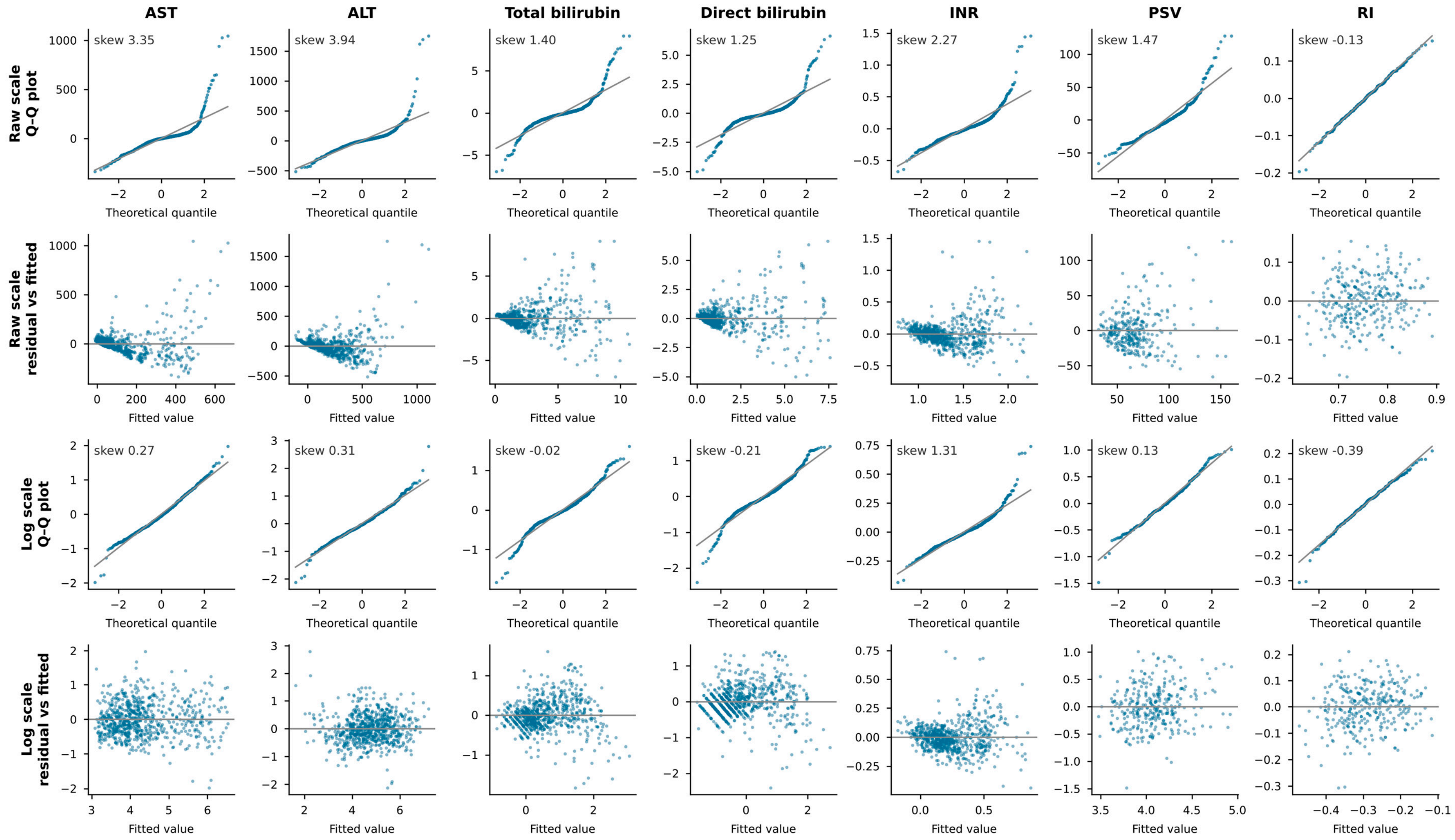

Supplement: Supplementary file 1 [file medicina-62-02006-s001.zip › medicina-4570357-supplementary.pdf]
